# Second- and third-generation ALK inhibitors for non-small cell lung cancer

**DOI:** 10.1186/s13045-016-0251-8

**Published:** 2016-03-08

**Authors:** Jingjing Wu, John Savooji, Delong Liu

**Affiliations:** Department of Oncology, The First Affiliated Hospital of Zhengzhou University, Zhengzhou, China; Department of Medicine, Westchester Medical Center and New York Medical College, Valhalla, NY 10595 USA

## Abstract

Crizotinib as the first-generation ALK inhibitor has shown significant activity in *ALK*-mutated non-small cell lung cancer (NSCLC). Second- and third-generation ALK inhibitors are entering clinical applications for *ALK*+ NSCLC. In addition, a third-generation ALK inhibitor, lorlatinib (PF-06463922), was reported to resensitize NSCLC to crizotinib. This review provided a summary of clinical development of alectinib, ceritinib, brigatinib (AP26113), and lorlatinib.

## Background

Small molecule inhibitors of EGFR (epidermal growth factor receptor) have been widely used for lung cancer therapy [1–9]. A small subset (3–13 %) of non-small cell lung cancer (NSCLC) has been shown to have rearrangements in the *ALK* (anaplastic lymphoma kinase) gene [10, 11]. Over the last few years, ALK inhibitors have shown significant benefits in the management of ALK-positive NSCLC compared to conventional chemotherapy [12–15]. A big caveat however is the emergence of resistance to ALK inhibitors [16]. This article provided a summary of clinical development of alectinib, ceritinib, brigatinib (AP26113), and lorlatinib for NSCLC.

## *ALK* gene and the roles in oncogenesis

**Fig. 1:**
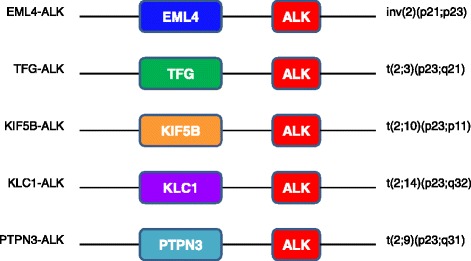
ALK mutations in non-small cell lung cancer. Most mutations of the ALK gene are in the form of a translocation with another partner gene leading to a fusion oncogene. Most common fusion oncogenes in non-small cell lung cancer are presented in this diagram

The *ALK* gene encodes for ALK receptor tyrosine kinase enzyme. The gene is located on the short arm of chromosome 2 (2p23) and belongs to the insulin receptor superfamily. Like other receptor tyrosine kinases, it has an extracellular domain, a transmembrane segment, and a cytoplasmic receptor kinase segment [17]. Physiologically, ALK is involved in the development of brain and neurons [18]. It is highly expressed during embryogenesis and thereafter becomes dormant. ALK mutation can lead to tumorigenesis [19]. Most mutations of the *ALK* gene are in the form of a translocation with another partner gene leading to a fusion oncogene which becomes overtly expressed in cancers [20] (Fig. 1). The first ALK mutation was reported in 1994 when *NPM*-*ALK* was described in a subset of anaplastic large cell lymphomas [21]. This mutation involves fusion of the nucleophosmin (*NPM*) gene and *ALK* as a result of t(2; 5) (p23; q35) [21, 22]. Additional gene partners have been discovered in fusion oncogenes with *ALK* gene. A few examples are *TPM3*-t(1;2)(q25;p23), *TFG*-t(2;3)(p23;q21), *CLTCL1*-t(2;17)(p23;q23), and *ATIC*-inv(p23;q35) [22]. More mutations of *ALK* gene have been reported in several cancers, including NSCLC, inflammatory myofibroblastic tumors, diffuse large B cell lymphoma, colon cancer, renal cell carcinoma, breast carcinoma, esophageal cancer, and neuroblastoma [23].

ALK mutations were first described in NSCLC in 2007 when a subset (7 %) of Japanese patients were found to have echinoderm microtubule associated protein like-4 (EML4) rearrangement with ALK leading to a fusion oncogene *EML4*-*ALK* [24, 25]. This was due to an inversion rearrangement from inv(2) (p21;p23). As a result, EML4 replaces the extracellular and intramembranous parts of *ALK* and fuses with the juxta membranous part. The *EML4-ALK* gene induced tumor formation in nude mice [23, 24]. Due to different breakpoint on *EML4*, several variants of *EML4*-*ALK* mutation have been described [10, 26, 27]. *EML4*-*ALK* variants with differing frequencies are V1 (54.5 %), V2 (10 %), V3a/V3b (34 %), and V5a (1.5 %) [26, 27]. Rearrangements of the *ALK* gene with partner genes other than *EML4* have been described, namely, *KIF5B, KLC1, TFG, TPR, HIP1, STRN, DCTN1, SQSTM1, and BIRC6* [28] (Fig. 1).

*ALK* translocations result in increased tyrosine kinase activity leading to increased cell proliferation and survival and ultimately tumorigenesis. The ALK signaling pathways involve phospholipase Cγ (PLCγ), Janus kinase (JAK)–signal transducer and activator of transcription (STAT), PI3K–AKT, mTOR, sonic hedgehog (SHH), JUN-B, CRKL–C3G (also known as RAPGEF1), RAP1 GTPase, and MAPK signaling cascades [23].

## *ALK*^+^ NSCLC characteristics

*ALK*-positive NSCLCs are generally seen in non-smokers, occur at a younger age, and are mostly adenocarcinoma in histology [15]. They also seem to have a female gender predisposition [11–13, 27]. Pathological features include solid morphology and presence of signet ring cells [29, 30].

## Crizotinib (PF-02341066, xalkori)

Crizotinib (PF-02341066, xalkori) is the first-generation ALK inhibitor approved for *ALK*-positive NSCLC [12]. It has a IC_50_ against *EML4*-*ALK* of 250–300 nm [31]. In addition to having activity against *ALK*, it also has activity against c-MET and ROS1 tyrosine kinases [31–34]. It was approved for *ALK* positive, locally advanced, and metastatic NSCLC [35].

The PROFILE 1007 study involving 347 patients with *ALK*-positive NSCLC compared crizotinib with chemotherapy in patients who failed at least one prior platinum-containing regimen [13]. These patients were randomly assigned to receive either 250 mg twice daily of oral crizotinib vs intravenous pemetrexed or docetaxel. The median PFS was 7.7 months (95 % CI 6.0–8.8) in crizotinib group compared with 3.0 (95 % CI 2.6–4.3) months in the chemotherapy group. The ORR was 65 % (95 % CI 58–72) in crizotinib compared to 20 % (95 % CI 14–26) in the chemotherapy group (*P* < 0.001) [13]. The adverse events reported were mostly grades 1 or 2. Grade 3 or 4 events were elevated aminotransferase levels and neutropenia which occurred in 16 and 13 % of patients, respectively [13, 15].

PROFILE 1014 study compared crizotinib vs chemotherapy in 343 patients who had no previous treatment for advanced NSCLC. They were randomized to either receive crizotinib vs pemetrexed plus platinum (cisplatin or carboplatin). Progression-free survival for crizotinib group (*n* = 172) was 10.9 months and for chemotherapy group (*n* = 171) was 7.0 months. The ORR was 74 % (95 % CI 67–81) for crizotinib group vs 45 % (95 % CI 37–53) for chemotherapy (*P* < 0.001). Median OS was not reached in either group at the time of report (hazard ratio for death with crizotinib, 0.82; 95 % CI, 0.54 to 1.26; *P* = 0.36); the 1-year estimated survival was 84 % with crizotinib vs 79 % with chemotherapy. Crizotinib-associated AEs were vision disorders, diarrhea, nausea, and edema. It was concluded from PROFILE 1014 study that crizotinib was superior to standard first-line pemetrexed-plus-platinum chemotherapy in patients with previously untreated advanced *ALK*-positive NSCLC. Hence, crizotinib is currently approved for first line in *ALK*+ NSCLC [14, 36].

Crizotinib has also been shown to be highly efficacious in ROS1-positive NSCLC which comprises 1 % of all NSCLC. In a phase 1 study of 50 patients, the ORR was 72 % (95 % CI 58–84) (33 PR and 3 CR). The median PFS was 19.2 months [31]. Among 30 tumors that were tested, 7 *ROS1* fusion partners were identified, 2 of these partner genes were novel. However, there was no correlation between the type of *ROS1* rearrangement and the clinical response to crizotinib. *ROS1* rearrangement molecularly marks a small subgroup of NSCLC for which crizotinib can play an active role in clinical therapy.

## Limitations of crizotinib

### Resistance to crizotinib

Majority of patients develop resistance to crizotinib within 1 to 2 years from the initiation of therapy [37]. The resistance to ALK inhibitors can be classified into primary and secondary resistance [38].

Primary resistance is seen when the tumor is deemed refractory to the agent at the beginning of the therapy itself as reported in chronic myeloid leukemia [39]. In the case of *ALK+* NSCLC, the primary resistance can be attributed to the different fusion variants of *EML4* with *ALK* or other partner genes [38]. Different sensitivities to crizotinib have been shown to be dependent upon the *ALK* variant or fusion gene partner [40, 41]. Currently, FISH has been the gold standard for detecting ALK mutations in NSCLC.

Secondary resistances are acquired mechanisms after the tumor has been exposed to an ALK inhibitor and can be further classified into two categories: ALK dominant and ALK non-dominant. In the ALK dominant type, there is mutation in the target *ALK* gene resulting in inability to inhibit the encoded tyrosine kinase. These are termed as ALK dominant as they depend upon ALK tyrosine kinase activity [42]. Most of the mutations are in the form of point mutations and the first ones to be described are C1156Y and L1196M [43]. There have been several other secondary point mutations that have been identified and are the following: G1269A, F1174L, 1151Tins, L1152R, S1206Y, I1171T, G1202, D1203N, and V1180L [41–44].

The *ALK* non-dominant resistance involves emergence of bypass tracks such as *EGFR* mutation, *KRAS *mutation, amplification of *KIT*, phosphorylated amplification of *ErbB, MET*, and activation of *IGF-1R* in the downstream signaling. It has been shown that in the same *ALK* resistant tumor, multiple mechanisms of resistances may occur [42, 45].

Secondary mutations of the *ALK* gene result in 29 % of resistant cases, and gene amplification is implicated in 9 % of these cases. The remaining of the cases can be attributed to bypass pathways and other mechanisms that have yet to be defined [46].

## CNS metastasis

Crizotinib has poor activity against CNS metastasis in NSCLC as evidenced by low concentrations detected in CNS samples during the course of systemic chemotherapy. The ratio of CNS to serum concentration of crizotinib has been in the range of 0.0006–0.001 as established by individual case reports [47–49]. In a retrospective analysis of trials involving crizotinib, 20 % of patients who did not have CNS disease at the beginning had CNS metastasis while on therapy [50]. PF-06463922 (lorlatinib) is a newly developed ALK inhibitor that has been designed for better CNS penetration and is currently in phase I/II trials (NCT01970865) (see below) [15].

In another analysis of 90 patients with brain metastases from *ALK*-mutated NSCLC, 84 of 90 patients received radiotherapy to the brain, and 86 of 90 received TKI therapy [51]. Significant improvement in this population of poor-prognostic patients was reported. The median OS after development of brain metastases was 49.5 months (95 % CI, 29.0 months to not reached), and median intracranial PFS was 11.9 months (95 % CI, 10.1 to 18.2 months). Four groups of patients were classified in this analysis with distinct outcomes: absence of extracranial metastases, high Karnofsky performance score ≥90, and no prior therapy with TKIs before development of brain metastases had longer survival (*P* = .003, <.001, and <.001, respectively), whereas isolated brain metastasis or initial treatment with radiation were not (*P* = .633 and .666, respectively). It was concluded that brain radiotherapy and TKIs to control intracranial disease in *ALK*+ NSCLC can lead to prolonged survival. Newer TKIs are playing an important role in this population of patients.

## Crizotinib toxicity

There have been case reports of significant adverse effects that were not reported in the initial trials. These included erythema multiforme [52], acute interstitial lung disease [53, 54], renal polycytosis [55–57], contact esophagitis [58, 59], decrease in GFR, and hypersensitivity reactions [14].

## Second-generation ALK inhibitors

### Ceritinib

Within the first year or two after crizotinib treatment is initiated, resistance typically arises. As mentioned above, mechanisms commonly include secondary mutations within the ALK tyrosine kinase domain and activation of alternative signaling pathways. More potent and structurally different inhibitors are therefore developed.

Ceritinib (LDK378, zykadia) is a potent ALK inhibitor compared to crizotinib [60–62]. A phase I study with 130 patients with *ALK*-positive advanced tumors included 122 NSCLC [63]. The doses were 50 to 750 mg in the dose escalation phase which enrolled 59 patients. The MTD of ceritinib was shown to be 750 mg daily. The dose-limiting toxicities (DLT) were diarrhea, vomiting, dehydration, elevated aminotransferase levels, and hypophosphatemia. Seventy-one patients received ceritinib in the dose expansion phase. One hundred fourteen patients received ceritinib dose of at least 400 mg daily. The ORR was 58 % (95 % CI 48–67). Among the 80 patients who failed crizotinib, the response rate was 56 % (95 % CI, 45 to 67). Among patients with NSCLC who received ceritinib with doses 400 mg or higher, the median PFS was 7.0 months (95 % CI, 5.6 to 9.5).

Thus, this study proved that ceritinib induced high responses in patients who failed crizotinib. Ceritinib was approved for treatment of relapsed or refractory NSCLC after crizotinib [64] (Table 1).

**Table 1:** FDA approved ALK inhibitors for non-small cell lung cancer

Trials	Patients	Agents	No.	ORR	PFS	*P* value	Refs.
Phase III PROFILE1007	Relapsed refractory	Crizotinib vs pemetrexed/docetaxel	173 174	65 % 20 %	7.7 ms 3.0 ms	<0.001	[13]
Phase III PROFILE1014	Untreated	Crizotinib vs chemotherapy	172 171	74 % 45 %	10.9 ms 7.0 ms	<0.001	[36]
Phase I	Failed chemotherapy and crizotinib	Ceritinib (NSCLC, ≥400 mg)	114	58 %	7.0 ms	N/A	[63]
Phase I/II	Failed chemotherapy and crizotinib	Alectinib (phase II)	46	94 %	NA	N/A	[67]
Phase I/II	Failed chemotherapy and crizotinib	Alectinib (phase II)	47	55 %	NA	N/A	[68]

*Abbreviations*: *N/A* not applicable, *No.* number, *ORR* overall response rate, *DOR* duration of response, *PFS* progression-free survival, *Ref* reference, *wks* weeks, *ms* months

### Alectinib

Alectinib (CH5424802, alecensa) is a potent and highly selective inhibitor of ALK tyrosine kinase with IC_50_ of 1.9 nM [65, 66]. More importantly, it has activity against L1196M which is one of the commonly seen secondary mutations in ALK gene leading to resistance to crizotinib.

In a multicenter, single-arm, open-label phase 1–2 study conducted in Japan (AF-001JP), ALK inhibitor naïve patients who had ALK-positive NSCLC were treated with alectinib [67]. In the dose escalation phase which included 24 such patients, increasing doses in the 20–300 mg range were used. No dose-limiting toxicities (DLT) were noted. Hence, 300 mg twice daily was established as the recommended dose for phase II. The phase II portion enrolled 46 patients. Forty-one of these patients had PR and 2 had CR. Hence, ORR was around 94 % (95 % CI: 82-98). Grade 3 adverse events were reported in 26 % (*n* = 12) and included elevated creatinine phosphokinase and neutropenia [67].

In another phase 1–2 single-arm open-label study, 47 patients with ALK-positive NSCLC who had resistance to crizotinib or were intolerant were treated with alectinib [68]. In the dose escalation phase, doses were escalated from 300 to 900 mg in seven different cohorts of patients. DLTs were seen in the 900-mg cohort: grade 3 headache in one patient and grade 3 neutropenia in another one. Three patients dropped out of the study due to adverse events: grade 3 dyspnea, grade 4 CNS metastasis, and grade 3 abdominal pain. Out of the 47 patients, 44 were assessed for response and the ORR was found to be 55 % (24 PR, one CR). ORR for the 21 patients who had baseline CNS metastasis was 52 % (5 CR and another 6 having partial CNS response). Therefore, this study showed that alectinib not only was effective in patients pretreated with first-generation ALK inhibitor but also was active for CNS metastasis [68]. Alectinib is now FDA-approved for the treatment of metastatic *ALK*+ NSCLC in patients who have progressed on or are intolerant to crizotinib.

## Brigatinib (AP26113)

Brigatinib is another second-generation ALK inhibitor. It is a potent dual inhibitor of ALK and EGFR, including *ALK* L1196M and EGFR T790M mutants, shown in preclinical and first-in-human studies [69–71]. In the initial dose-finding study, there were 18 evaluable *ALK*+ pts. Among these patients, 10 responded. Fifteen *ALK*+ pts had 0 (*n* = 3) or 1 (*n* = 12) prior ALK TKI (crizotinib); of these, 8/12 pts (67 %) responded, including two complete responses. Radiographic improvement was seen in 4 of 5 *ALK*+ pts with untreated or progressing CNS lesions. There were 16 pts enrolled with *EGFRm* history (15 NSCLC, 1 SCLC); 14 pts had ≥1 prior EGFR TKI. Of 12 EGFRm pts with a follow-up scan, 1 pt (prior erlotinib) responded at 120 mg, 6 pts had stable disease.

In the last update of the phase I/II single-arm, open-label, multicenter study in patient pts with advanced malignancies (NCT01449461), patients received brigatinib as the following: phase I: 30–300 mg/day total daily dose; phase II: 90 mg/day, 180 mg/day, or 90 mg/day for 7 days followed by 180 mg/day. Safety was reported in all 137 treated pts; efficacy was evaluated in all 79 *ALK*+ NSCLC pts [72] (Table 2). Most common treatment-emergent adverse events (TEAE) included nausea, diarrhea, fatigue, cough, and headache. Early-onset pulmonary events were observed less frequently with the 90-mg starting dose compared with higher doses. Median progression-free survival (PFS) is 56 weeks, 47 weeks with prior crizotinib. In pts with baseline CNS metastases, half of 12 pts had a brain response and 8/26 pts with only non-measurable lesions had disappearance of all lesions. Median intracranial PFS for these pts is 97 weeks. Therefore, brigatinib was active in crizotinib-resistant NSCLC and showed activity in CNS lesions. A randomized phase 2 trial of brigatinib in crizotinib-resistant *ALK*+ NSCLC (ALTA) is underway.

**Table 2:** Brigatinib and lorlatinib in clinical development for non-small cell lung cancer

Trials	Agents	Patients	No.	ORR	PFS	Refs.
Phase I/II (NCT01449461)	Brigatinib	Crizotinib-naive Crizotinib-failure with CNS metastases	7 65 38	7/7 (100 %) 45/65 (69 %) 14/38 (37 %)	56 weeks 47 weeks 97 weeks	[72]
Phase I/II (NCT01970865)	Lorlatinib	Untreated Failed 1 ALKi	N/A	N/A	N/A	[77]

*Abbreviations*: *N/A* not applicable, *ORR* overall response rate, *DOR* duration of response, *PFS* progression-free survival, *Ref* reference, *wks* weeks, *m* months, *ALKi* ALK inhibitor

## Third-generation ALK inhibitor

Lorlatinib (PF-06463922) is a novel, reversible, potent ATP-competitive small molecule inhibitor of ALK and ROS1. This third-generation inhibitor is effective against all known resistant mutants [73–75]. In preclinical studies, lorlatinib was proven to be active in crizotinib-resistant cancers both in vitro and in xenograft models [73–75]. To overcome *ALK* mutations and ALK inhibitor resistance, lorlatinib was combined with PI3K pathway inhibitors, such as PF-05212384 (PI3K/mTOR), GDC0941 (pan-PI3K), or GDC0032 (beta-sparing). Such rational combination was reported to lead to more robust activity in vitro and greater duration of efficacy in vivo in the ALK inhibitor resistant models [76].

Lorlatinib is being studied in a phase I clinical trial in patients who were refractory to crizotinib and ceritinib (NCT01970865) [77]. One patient enrolled to this trial responded to lorlatinib for 8 months. Interestingly, the patient was resensitized to crizotinib after the patient failed the lorlatinib treatment, indicating that retreatment under molecular guidance can be a clinically meaningful approach.

## Conclusions

Second- and third-generation ALK inhibitors are entering clinical applications for *ALK*+ NSCLC. Among these, dual inhibitors targeting ALK as well as EGFRm and ROS1 may provide additional benefits for crizotinib-refractory patients. Resensitization to and retreatment with crizotinib can be considered under molecular guidance. More and more biomarker-targeted agents are entering clinical applications [78–81]. Immune therapies are showing remarkable benefits [82–91]. It is foreseeable that combination of these novel agents and small molecular inhibitors may expand the potential for treatment of refractory lung cancer patients.
